# ‘A life I can cope with’. An alternative model of cognitive behavioural therapy (CBT) for CFS/ME

**DOI:** 10.1111/hex.13326

**Published:** 2021-09-02

**Authors:** Catherine Clark, Sue Holttum

**Affiliations:** ^1^ Salomons Institute for Applied Psychology Canterbury Christ Church University Canterbury UK

**Keywords:** chronic fatigue syndrome/ME, cognitive behavioural therapy, grounded theory, therapeutic processes

## Abstract

**Objectives:**

This study aimed to explore the experience of cognitive behavioural therapy (CBT) aimed at better management of chronic fatigue syndrome/myalgic encephalomyelitis (CFS/ME), rather than increasing activity.

**Design:**

This was a qualitative study using grounded theory analysis.

**Methods:**

Semi‐structured interviews were conducted with 13 adults who had engaged in CBT at a specialist CFS/ME service in which CBT is aimed at improved management of the condition.

**Results:**

A model was produced in which participants felt more able to cope with CFS/ME. Reduced fatigue did not seem to be a necessary precondition to managing. This has implications for CBT for CFS/ME.

**Conclusions:**

Specialist CBT for CFS/ME may result in improved coping and reduced distress, independently of changes in fatigue.

**Patient or Public Contribution:**

The researcher met with a representative from the university's service user advisory group (SAGE), who had lived experience of CFS/ME. They commented on possible questions for the interview topic guide and provided advice on ways in which ethical issues specific to CFS/ME could be considered, for example, prevention of harm during interviews. Second, for quality assurance as part of respondent validation, a proposed draft of the grounded theory was discussed with participants.

## 1 BACKGROUND

In the United Kingdom, the National Institute for Health and Care Excellence^1^ recommends that people with chronic fatigue syndrome/myalgic encephalomyelitis (CFS/ME) of all severities be offered cognitive behavioural therapy (CBT). The 2007 guidance states that CBT for CFS/ME should incorporate some components aimed specifically at reducing the core symptom of fatigue, including supporting gradual increases in activity and ‘challenging thoughts and expectations that may affect symptom improvement’. The guidance states that CBT should also include more generic CBT components aimed at helping people adapt to illness, such as learning assertiveness skills to help people set limits on their activity, and supporting adjustment to the diagnosis. The recent 2020 draft National Institute for Health and Care Excellence (NICE) guidance represents a shift from the 2007 guidance,^1^ highlighting that CBT is not a treatment or cure for ME/CFS, but may be useful in supporting people who live with ME/CFS to manage their symptoms.^2^

A Cochrane meta‐analysis^3^ reported that CBT was more effective than usual care and other psychological therapies for reducing fatigue in adults with CFS/ME, with 40% of participants showing improvement after CBT compared with 26% in usual care. However, a large patient survey conducted by the UK's M.E. Association found that between 8% and 35% of participants reported improvement after CBT and 18% reported adverse effects.^4^ Yet, there has been considerable publicity on how effective CBT is for CFS/ME. Friedberg^5^ suggests that this may underpin the considerable backlash against CBT from patients, much of which has been on social media. The new draft NICE Guidance^2^ recommends CBT as a form of support for self‐management of CFS/ME, but does not suggest that it will eliminate difficulties.

It has been highlighted that the fundamental treatment goals for CBT for CFS/ME vary across studies.^6^ Some CBT interventions, such as those investigated in the high‐profile PACE trial,^7^ adopt a model that aims to increase activity to promote recovery. This is based on the ‘cognitive behavioural model of CFS’,^8^ which suggests that CFS/ME is maintained by unhelpful beliefs about exercise and illness, for example, ‘This activity will make me feel worse’ (p. 537), which result in activity avoidance and subsequent changes in physiology such as loss of muscle strength, collectively known as ‘deconditioning’. The cognitive theory suggests that reconditioning can lead to recovery from the condition. Another model of CBT for CFS/ME aims to instead support people to adapt to the condition and find an optimal level of activity to minimise exacerbation in symptoms.^6^ Most CFS/ME patients do not believe that their illness is psychogenic and can be cured by increasing their activity, although many acknowledge that movement may play some role in recovery.^9^, ^10^ The M.E. Association has advocated that interventions focused on better management of the condition are more useful for CFS/ME than interventions whose primary goal is an increase in activity. Yet, despite this, many people with CFS/ME are offered CBT focused primarily on increasing activity.^10^

NICE^1^ highlights that one aim of CBT should be to reduce the ‘distress associated with CFS/ME’. Yet, CFS/ME intervention studies have typically focused on fatigue and functioning as primary outcome measures, rather than changes in distress. The available evidence on psychological outcomes for CBT for CFS/ME is mixed. Price et al.^3^ found that CBT failed to reduce distress in comparison to usual care. However, a meta‐analysis by Castell et al.^11^ showed that following CBT, participants experienced a significantly greater reduction in depression and anxiety than controls. As yet, there are no studies aimed at understanding processes via which a reduction in psychological distress may follow from CBT for CFS/ME. Brooks et al.^12^ found that after CBT, patients showed significantly increased acceptance of the condition and reductions in two aspects of perfectionism (concern over mistakes and doubts about actions). It was found that increased acceptance post‐CBT was significantly correlated with improvement in fatigue, physical functioning and work and social adjustment.

However, research exploring *psychological* change following CBT for CFS/ME is in its infancy and the specific perceived relationships between coping, acceptance, fatigue and other factors are yet to be explored. In addition, there are very few studies exploring models of CBT with the primary goal of supporting participants to live with CFS/ME, rather than increasing their activity to reduce fatigue. Yet, this former model is being offered by services and warrants further investigation.

This study therefore sought to build a preliminary model of therapeutic change for CBT aimed at better adjustment to and management of CFS/ME, exploring the inner workings of the model and ways in which it may effect change.

## 2 METHODS

### 2.1 Design

This study was qualitative and used interviews.

### 2.2 Participants

Participants (*n* = 13) had all been assessed as fulfilling the criteria for a diagnosis of CFS or ME by a doctor in the service. The service adopted a pragmatic approach to diagnosis, in which clinicians used more than one set of diagnostic criteria. The median age of the participants was 49 (range: 19–72). As shown in Table 1, six participants were in paid work, one in education, five were retired or unemployed and one was a home‐maker. Participants reported attending between 6 and 10 sessions of specialist CBT at the service (median = 6). Three participants had previously undertaken additional episodes of specialist CBT for CFS/ME at other services. They reported that, as within the service, the goal had been positioned as better management of CFS/ME; therefore, data relating to these experiences were included in the analysis. Change scores were not noted for this study, but participants mainly reported lack of change on physical symptoms. The lack of questionnaire data is revisited in Section Results, 4.

**Table 1:** Participant demographic information

Pseudonym	Age	Gender	Ethnicity	Employment status when starting CBT	Episodes of CBT for CFS/ME
George	57	M	White British	Medically retired	One episode
Sarah	51	F	White British	Home‐maker	Three episodes (1 at ‘the service’; 2 at another specialist service)
John	42	M	White British	Unemployed	One episode
Jean	45	F	White British	Unemployed	One episode
Charlie	56	M	White British	Full‐time work	Two episodes (1 at ‘the service’; 1 at another specialist service)
Eva	29	F	White British	Full‐time work	One episode
Susan	53	F	White British	Medically retired	One episode
Judith	72	F	White British	Retired	One episode
Layla	19	F	Mixed Black, Asian and White European	In education	One episode
Will	35	M	White British	Part‐time work	One episode
Sebastian	56	M	Black British	Full‐time work	Two episodes (1 at ‘the service’; 1 at another specialist service)
Rachel	29	F	White British	Part‐time work	One episode
Fiona	49	F	White British	Part‐time work	One episode

Abbreviations: CBT, cognitive behavioural therapy; CFS/ME, chronic fatigue syndrome/myalgic encephalomyelitis; F, female; M, male.

### 2.3 Procedure

Participants were recruited through an NHS specialist community CFS/ME service. In contrast to the CBT models adopted in many CFS/ME services,^11^ the model of CBT delivered in this service was not one with the primary aim of increasing activity to facilitate ‘reconditioning’ and a reduction in fatigue. Instead, the primary goal was increased quality of life by better adjustment to and management of CFS/ME. The therapists did not follow a manual or use a specific model of CBT for CFS/ME, but rather drew on a range of CBT techniques and concepts, both specific to CFS/ME (e.g., pacing) and more general (e.g., lowering standards, assertiveness, self‐soothing), tailoring the therapy to each individual. A reduction in fatigue was an anticipated secondary benefit for the intervention. CBT was delivered by two CBT therapists in the service who had been qualified for 7 and 11 years, respectively.

Following ethical approval, the service contacted all service users who had completed CBT within the previous 2 years about the study by letter. Participants were given an information sheet containing information about confidentiality, risk and the right to withdraw from the study at any time. Individual semi‐structured interviews lasted between 52 and 93 min. Six interviews were conducted in person in a research space adjacent to the service and seven were conducted over Skype. All interviews were conducted by the first author, a female trainee clinical psychologist aged between 30 and 40 years with previous experience of conducting research interviews at the doctoral level. The interviewer was not associated with the service and was not known to the participants. Interviews were audio‐recorded and transcribed. Analysis was primarily conducted by the first author.

### 2.4 Data analysis

Analysis began after the first interview and continued concurrently with data collection. It was informed by the approach described by Strauss and Corbin.^13^ Constant comparison and memoing were used throughout to identify and record similarities and differences between data from different participants and to aid category development. Theoretical sampling^14^ was achieved by adapting interview questions according to emerging categories within the data.

Analysis began with detailed line‐by‐line open coding aimed at identifying concepts within the data. It then progressed to axial coding, exploring the properties and dimensions of categories. Data were then analysed for context, keeping in mind the concept of conditional/consequential matrices.^13^ Relationships between categories were explored by creating maps of each participant's experience. The later stages of analysis involved selective coding and broader theoretical integration. In the final stages, constant comparison highlighted that new data reflected existing major categories in the model and that these categories were dense in terms of properties and well integrated, suggesting theoretical saturation.^13^ Dimensional variation was more limited due to the absence of participants who reported poorer outcomes from the intervention.

### 2.5 Quality assurance

Elliott et al's.^15^ quality guidelines were used to increase the credibility of the research. A research diary was maintained, a bracketing interview was conducted and a positioning statement was created. Two line‐by‐line coded transcripts, memos around emerging categories and integrative maps were shared with the second author, who offered comments, challenges and elaborations, which were discussed and incorporated into the analysis. The second author, like the first, had no connection with the service. Finally, respondent validation was undertaken to ascertain the credibility of the analysis to participants.^16^ A proposed draft of the theory was sent to participants and discussed via phone or email.

## 3 RESULTS

**Figure 1:**
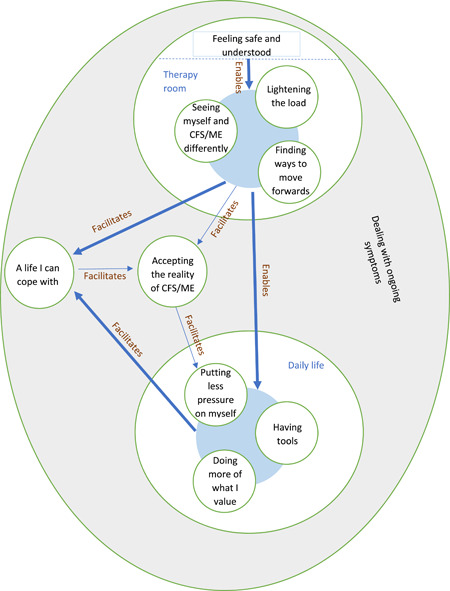
Model of theory. CFS/ME, chronic fatigue syndrome/myalgic encephalomyelitis

As shown in Table 2, 10 main categories were developed from the data. The categories are illustrated in the model in Figure 1. The principal context of the therapy experience was *dealing with ongoing symptoms*. Four categories describe experiences in the therapy room (illustrated in the top circle in Figure 1) and three categories describe experiences in daily life (illustrated in the bottom circle in Figure 1). As illustrated by the shaded circles in Figure 1, there was often interaction between the different experiences within each context. Experiences in the therapy room and daily life seemed to lead to changes in the meaning that participants held around their lives with CFS/ME, as reflected in the categories ‘A life I can cope with’ and ‘Accepting the reality of CFS/ME’. Each of the categories and their subcategories will be discussed, with emphasis on the relationships between them. Participants have been assigned pseudonyms to maintain anonymity.

**Table 2:** Categories and subcategories identified in the data

Context	Category	Subcategory
Not specific to one context	Dealing with ongoing symptoms	Lack of improvement from previous interventions
		Minimal changes over CBT
Therapy room	Feeling safe and understood	
	Seeing myself and CFS/ME differently	CFS/ME is real and not my fault
		CFS/ME is manageable
		Lowering expectations of myself
		It's ok to put myself first
	Finding ways to move forwards	Understanding what was happening
		Separating what we can and cannot control
		Learning tools and problem solving
		Finding opportunities for enjoyment
	Lightening the load	Resolving broader issues
		Getting things off my chest
Not specific to one context	A life I can cope with	Having ways to take control
		Getting my confidence back
		Living beside CFS/ME
		Making peace with CFS/ME
Not specific to one context	Accepting the reality of CFS/ME	
Daily life	Having tools	Taking a different perspective
		Control over my responses
		More able to communicate
	Putting less pressure on myself	Cutting back on demands
		Reprioritizing my needs
		Being more open about my needs
	Doing more of what I value	More courage to do things
		Having more capacity

Abbreviations: CBT, cognitive behavioural therapy; CFS/ME, chronic fatigue syndrome/myalgic encephalomyelitis

### 3.1 Categories

#### 3.1.1 Dealing with ongoing symptoms

In the first of two subcategories, participants described *lack of improvement from prior interventions*. Despite engaging in a range of interventions before CBT, all but one participant continued to experience significant CFS/ME symptoms. Jean was an exception as she felt ‘85% back to normal’ when starting specialist CBT, which she attributed to graded exercise:

The weird thing about ME is that the only cure seems to be, you know, the graded exercise. (Jean)

Participants also described experiencing *minimal changes over CBT*:

Most participants experienced no change in their CFS/ME over CBT:

I don't think in any way it helped my ME, but it did help my mind. (Sarah)

Those reporting some improvement did not return to pre‐illness levels, but described fewer relapses:

I don't want to say reduced the symptoms, but probably have slightly fewer relapses and maintain a slightly better level quality of life. (Susan)

The next four categories pertain to experience of the therapy room.

#### 3.1.2 Feeling safe and understood

Feeling safe, contained and believed allowed participants to open up:

I cried quite a lot, quite a lot in the therapy room and it felt like it was okay to do that. (Sarah)

Making progress seemed more likely when participants felt that the therapist adopted a holistic approach and understood their unique difficulties:

It wasn't just focused on ME. It was looking at my whole body or mind and seeing how I was coping with things, so I was actually really pleased. (Susan)

Participants were consistently told that the goal of therapy was better management of CFS/ME. All participants found this suitable as it fitted with their experience of the condition.

#### 3.1.3 Seeing myself and CFS/ME differently

There were four subcategories, first: *CFS/ME is real and not my fault*.

For several participants, therapists highlighting that CFS/ME was a real and chronic condition gave participants a clearer representation of the illness and its impact:

I sort of had somebody else say, ‘well no you are quite right, you shouldn't be doing that [activity]’. (George)

For some participants, exploring the causes of CFS/ME reduced frustration and guilt:

I think it stopped me using energy that I don't have, being angry about it and looking ‘why has this happened?’ (John)

Therapists provided hope that *CFS/ME is manageable*, which reduced fear of it:

She made me realise that it's just something in my back pocket, it doesn't have to be something that completely controls my life. (Layla)

Many participants realised that they placed high expectations on themselves and were able to embrace *Lowering expectations of myself*.

Saying like…if you went to somebody's house, would it matter if you saw them, or would it matter if they produced loads and loads of food? (Susan)

Participants increasingly recognised that *It's OK to put myself first*, often resolving ambivalence and guilt about responsibilities:

I came away just with that re‐focus on putting myself first and not being afraid to do that actually, it is okay to do that when you are not well. (Sarah)

This was often facilitated by direct therapist advice.

She [the therapist] basically said, ‘well just tell them to go away, you don't have to do anything you don't want to do’ and that was a… I don't know why I thought I did need to. (Jean)

#### 3.1.4 Finding ways to move forwards

Again, there were four subcategories. Participants found it helpful to identify factors exacerbating their symptoms and distress, including patterns of behaviour and thinking, that is, *Understanding what was happening*. This reduced stress and allowed them to respond differently.

Where everything's overwhelming and everything's impossible, just being able to take a look at each piece sort of individually…because there's so many different aspects to how you end up not being able to do anything. (Fiona)

Understanding what's going on, it makes such a difference in terms of actually being able to cope with it. (Will)

Participants valued *Separating what we can and cannot control*, which helped them to let go of some distress and worrying:

I think one of the biggest things for me that I remember was talking about what we kind of can and cannot control. (Rachel)

Therapists supported participants in *Learning tools and problem solving* skills, to apply to their broader lives:

I just mean like, dissect things. So, whereas before it'd just be like, ‘woah, I'm really stressed, and there's loads of stuff, and I can't cope’. (Rachel)

Concrete planning exercises helped participants look to the future and helped in *Finding opportunities for enjoyment*:

Rather than spending my entire life thinking about getting back to ‘so called normal’ and thinking about all the things I couldn't do and thinking what *can* I do and thinking how am I going to do it. (George)

#### 3.1.5 Lightening the load

In the first of two subcategories, participants came to experience less anxiety about other issues, such as personal traumas, relationships and identity, that is, *Resolving broader issues*:

So it was that sort of, always wanting to be loved, always being, wanted to be accepted, to the point of ‘I'm really not that fussed with it.’ (Sebastian)

This often resulted in feeling less need to change things, resulting in more capacity to manage their condition:

I do feel that I can clear the way to improvement by dealing with that [personal trauma]. (Judith)

Participants felt ‘lightened’ and ‘lifted’ after *Getting things off my chest*:

It was just a rare opportunity for me to open up to somebody and get things off my chest. (John)

#### 3.1.6 A life I can cope with

In the first of four subcategories of this category, participants described *Having ways to take control*, including understanding, ‘tools’, solutions and support.

I think it [saying no to people] made me feel more in control. (Jean)

Participants felt less vulnerable:

I don't care, because now I've got a plan B. (Charlie)

Participants experienced *Getting my confidence back*, which allowed them to engage more positively in work and personal contexts:

I'd go back to the pub. I started not to become paranoid, you know? (Sebastian)

They could be more compassionate to themselves about having the condition:

I stopped beating myself up, I was knocking myself. (John)

Adjustments to their outlook and lifestyle led participants to see CFS/ME as taking less away from their lives and themselves as *Living beside CFS/ME*:

feeling that it was something that wasn't necessarily going to ruin my life. (George)

I can live beside it rather than, it doesn't dominate me in the same way anymore. (Susan)

Life with CFS/ME could seem more compatible with values and desired roles:

I remember sort of coming out thinking ‘yeah okay I can work with that, you know, it is a good thing for them [my children]’. (Sarah)

This reduced the need for certainty and seeking further interventions:

Just putting it in your back pocket kind of makes it feel okay to know what's not going to happen in the future. (Layla)

Most participants felt less distressed and more hopeful about living with the illness, described as ‘*making peace with ME/CFS’* by Layla.

This life, I am living with it, it's helped me to be more relaxed with it. (John)

However, frustration about the condition remained for several participants:

It's difficult, it's not the mum I want to be. (Sarah)

#### 3.1.7 Accepting the reality of CFS/ME

Many participants underwent the process of ‘accepting’ having CFS/ME. There were no subcategories. This involved acknowledging the extent of their symptoms and absence of an immediate way of curing or controlling them, but did not mean relinquishing hope of gradual longer‐term improvement:

Just kind of accepting that at least for the time being. Yeah I think not thinking this is forever. (Will)

For some, acceptance happened before CBT, but many described CBT facilitating this:

Before I was fighting it in every way. This has just made me say, ‘yeah I am shattered’. (John)

Acceptance could result from didactic therapist approaches:

To have someone actually saying, ‘well when are you going to accept that that's what you've got?’, it's like, oh my god, it's quite a wake up. (Fiona)

Or participants being more compassionate to themselves:

not being so hard on myself. That's what made me accept I'm a CFS person. (Sebastian)

Acceptance became easier when participants saw CFS/ME as less detrimental, but was often a painful process:

And it was learning that I can grieve that I can't do dinner parties for sixteen people anymore, but actually that that doesn't matter. (Susan)

Sebastian reported only accepting the nature of CFS/ME after a second course of CBT.

#### 3.1.8 Having tools

This first of three categories relevant to daily life also has three subcategories.

Participants gained capacity for *taking a different perspective* on symptoms and, more broadly, often using metaphors or images to think about unhelpful thought patterns:

Just seeing it [symptom worsening] as like waves you know, that occasionally you get three big ones in a row… Really noticing when I am catastrophising for instance. (George)

This reduced distress:

It stopped me from spiralling to overwhelmed and despair a lot more. (Eva)

However, it was harder to challenge thinking when symptoms intensified:

I know I can generally pick up from these sort of dips but it is still difficult to convince yourself that it is a temporary blip. (Sarah)

Participants saw tools such as time out and problem solving as ways of gaining *control over my responses*, allowing them to do more of what they wanted:

Just by counting, ‘one, two, three, four, one, two, three, four’, that's really helped me be able to get up and take the next steps. (Will)

This also helped in managing their condition:

Whereas previously to that, I'd just kind of said yes [to requests]. (Rachel)

Having tools also entailed being *More able to communicate* for several participants, helping them to negotiate with others, particularly about their needs:

Just going from, ‘I'm tired all the time’, to having more vocabulary is definitely helpful. (Rachel)

This could lead to better support:

I think with personal understanding comes your circle become more understanding as well. (Rachel)

Better communication could be due to feeling less preoccupied and distressed, having ‘lightened the load’ (see earlier) in the therapy room:

I could save the emotional stuff and dump it there and then be more choosey and more selective about what I ran past him [my husband]. (Sarah)

#### 3.1.9 Putting less pressure on myself

In the first of three subcategories, almost all participants mentioned *cutting back the demands* placed on themselves in work and social contexts. This appeared to be due to better understanding and acceptance of their condition and re‐evaluating their expectations of themselves:

Trying to actually have rest properly if you want to rather than waiting until you're just so wrecked you can't do anything. (Fiona)

I just have given up trying to achieve as much. (Susan)

Some initially required implicit ‘permission*’* (Rachel) from the therapist to cut back:

Others found it necessary to use strict boundaries.

It's now got different boxes, different sections. That's my work box, that's it, gone, nine to five. (Sebastian)

Cutting back was less likely for those who had not reached an understanding of the condition:

It [CBT] wasn't long enough for me, myself, to reach an understanding of what I needed to do to protect myself. (Judith)

Lack of acceptance of the chronic nature of the condition could result in reverting to old habits:

I thought I was cured. You know, and I carried on, I thought ‘yeah, it's back to going to events and weekends away’. (Sebastian, first course of CBT)

For several participants, re‐evaluating their responsibilities led to *Re‐prioritising my needs*:

I am being more selfish about making those decisions and if I can throw money at the issue I will throw money at the issue and if I can't then someone else has to help out. (Sarah)

This shift was often uncomfortable, but became easier with experience:

Once I started to actually say something, it wasn't negative, I haven't lost my job, I haven't gone down the ladder. (Rachel)

Participants often introduced new forms of self‐care, often based on therapist advice:

I'll go make myself a cup of tea or something. So yeah, I more sort of made myself a bit more important. (Layla)

For many participants, reducing pressure on themselves meant *Being more open about my needs*, whether physical or emotional:

For her [my wife] to be told that she was impacting on my health was quite a difficult conversation. (Sebastian)

This could become easier after the therapist provided validation and a positive experience of discussing the condition:

I suppose because someone else has validated it, rather than feeling like, it's very difficult to say to someone ‘actually I just feel really really shit’. (Fiona)

Increased openness typically led participants to feel more supported:

They are a little bit more aware and a little bit more understanding. (John)

#### 3.1.10 Doing more of what I value

In the first of two subcategories, participants described having *More courage to do things*:

I didn't let it [CFS/ME] kind of stop me doing things as much… (Will)

I'm more able to kind of like go back to the source rather than just living in that perpetual cycle of, ‘I'm just going to be really tired, so I can't do it, can't do it, can't do it.’ (Rachel)

Participants could also relax and engage more fully during activities:

Rather than doing it and questioning whilst I'm doing it, if I can do it. It's more like I'm doing it because I actually know I can do it. (Layla)

Participants felt less preoccupied and stressed, which meant *Having more capacity*.

for valued activities and topics of conversation.

Being able to kind of like sort things out from a work perspective and push back there, I was kind of able to go back to singing. (Rachel)

If I got it out of my system I would be more likely to talk about other stuff. (George)

Participants had more time and energy:

Being able to take time to maybe go to the travel agent with my wife or go on the website. Before I would be too tired to do that, I would just drop in bed. (Sebastian)

Activity planning exercises helped participants regain a sense of agency:

I suddenly started thinking, ‘okay I've got to maybe get my brain working again’ and ‘start thinking for myself’. (Jean)

#### 3.1.11 Results of respondent validation

The five participants who responded all broadly endorsed the results of the study. For example:

*I think that would be a useful handout to show people who are suffering symptoms the potential way forward. It all relates to how I was and am now. (Charlie)*

*Before this I kept having relapses, not realising that I was exacerbating the underlying condition by overdoing it. By rationing my energy, avoiding stress (vital!) and looking for inspiring and uplifting experiences I have made great progress, and CBT is to thank for it. As someone who has suffered from CFS I feel very strongly that this therapy must be made readily available. (George)*

## 4 DISCUSSION

Participants described intervention components from the therapy room, alongside subsequent changes made in daily life, as enabling them to cope better with life with CFS/ME. This echoes previous studies exploring CBT for CFS/ME,^17^, ^18^ although in these studies, the focus of the CBT was increasing activity.

In the present study, participants felt more in control and saw it as more possible to live alongside the condition. Partly, this seemed to result from addressing issues that were preventing participants from being able to manage their CFS/ME, such as holding themselves to high standards. This echoes Brooks et al.,^12^ who found reduced perfectionism and fatigue after CBT for CFS/ME. Brooks et al.^12^ questioned whether the reduction in perfectionism in fact reflected a reduction in ‘goal discrepancy’ (being unable to achieve a valued goal) as a knock‐on effect of reduced fatigue. The present study suggests otherwise, as participants described lowering expectations of themselves, often without any associated improvement in fatigue. In fact, data suggested that it was a lowering of standards that led to better management of symptoms, both directly and via increased acceptance of the condition.

This, together with learning tools such as problem solving and pacing, seemed to allow participants to approach their daily lives in a more adaptive way, preventing symptom flare‐ups and allowing more time and energy for valued activities. Participants valuing tools for managing energy or ‘pacing’ echoes the survey results presented in Geraghty et al.^4^ Participants felt more able to manage their daily lives after learning tools for managing emotions and resolving sources of distress in their broader lives, such as traumas or social anxiety. Participants felt more able to cope with CFS/ME after addressing anxieties about how CFS/ME prevented them from being able to fulfil their roles or values. Thus, as aimed for in CBT, results suggested a shift in both cognitions and behaviours. These were not, however, those cognitions and behaviours cited as key targets in the dominant cognitive behavioural models of CFS^7^, ^8^ that is, unhelpful illness beliefs and activity avoidance. CBT is an umbrella term, and our results confirm that multiple models of CBT are in use within the field of CFS/ME.

### 4.1 Acceptance

In line with studies of other psychosocial interventions for CFS/ME,^19^, ^20^, ^21^ including CBT,^12^ several participants reported greater ‘acceptance’ of having a chronic illness. They acknowledged that there was no immediate way of curing or controlling their symptoms, while retaining hope of gradual longer‐term improvement. Acceptance seemed to enable participants to further adjust their daily life, such as reprioritising their needs and putting less pressure on themselves. This echoes Pinxsterhuis et al.,^22^ who found that outside of interventions, the use of adaptive coping strategies in CFS/ME, such as pacing, was facilitated by participants accepting the reality of their condition and finding ways of rebuilding their identities.

Making adjustments in turn gave participants the experience that they could cope with a life with CFS/ME. This then appeared to promote further acceptance of the condition. This suggests a virtuous circle of acceptance, better coping and further acceptance. In their study of CBT, Brooks et al.^12^ suggested that acceptance may have been driven by improvements in fatigue, which resulted in the condition ‘interfering’ less in people's lives. The cycle highlighted in this study suggests that acceptance can be driven by a perceived improvement in *functioning*, even if participants do not perceive improved *fatigue*.

The study extends existing knowledge by identifying factors that seemed to facilitate this. For some, acceptance resulted from reduced guilt and anxiety after receiving information and/or validation that CFS/ME was not their ‘fault’. For others, acceptance resulted from letting go of high self‐expectations, which was described like ‘grieving’ a former identity. Rebuilt identities could then accommodate a representation of CFS/ME, allowing acceptance.

### 4.2 Limitations

This study did not attempt to represent the experience of all people with CFS/ME who have undertaken CBT. The sample size in this study was typical for qualitative studies, including those in the area of CFS/ME interventions, for example, Picariello et al.,^17^ but nonetheless relatively small, and it will be important for future research to explore the extent to which these findings are generalisable to a wider range of people with CFS/ME. Most participants seemed to have benefited from CBT; it may have been the case that service users who did not participate in the research had a less positive experience of the therapy. It would have been desirable to use purposive sampling to capture a breadth of experience, as those with poorer outcomes from CBT on standardised measures may have provided more data around barriers to therapeutic change; however, this was not possible due to limitations in recruitment. Ideally, it would have been helpful to have known the self‐reported change for each participant on such measures. However, posttherapy data were incomplete. A wider range of ethnic variation amongst participants would also have been desirable.

Although a strength of this study is the various ‘credibility checks’ that were put in place, participants' responses both during the interviews and respondent validation may have been influenced by a social desirability bias.

Lastly, in terms of replicability, the therapists at the service were not able to provide a therapy protocol. They described general issues often addressed in the therapy (e.g., lowering high standards, pacing), but were guided by clinical judgement to individualise therapy for each client. For this reason, it may be important in future studies to incorporate in‐depth interviews with therapists and video‐assisted analysis of therapy to more clearly understand therapeutic processes and link these with client‐reported experiences and outcomes, such as those described in this study.

### 4.3 Clinical implications

This study suggests explanations as to how CBT with the primary aim of improving quality of life rather than increasing activity and reducing fatigue can be experienced by service users as beneficial and result in reduced distress about life with CFS/ME. And that this can be achieved in a time‐limited and cost‐effective way (median six sessions). The results suggest two key areas on which this type of intervention should focus: First, by addressing barriers to service users accommodating CFS/ME in their daily lives and accepting the nature of the condition, for example, providing education and validation and supporting service users to lower their expectations of themselves and better prioritise their own needs. This should involve exploring service users' identities, roles and values and how these may be reconciled with CFS/ME. Second, interventions should provide cognitive and behavioural tools for managing anxiety about CFS/ME and its symptoms (e.g., challenging catastrophising, using grounding techniques) and managing the demands of daily living (e.g., problem solving, addressing worry by separating what can and cannot be controlled). Interventions should also teach pacing where appropriate.

It is important to consider this in the context of the NICE guidance.^1^, ^2^ The CBT model in this study did not focus on increases in activity despite the 2007 guidance recommending ‘Establishing a stable and maintainable activity level (baseline) followed by a gradual and mutually agreed increase in activity’.^1^ The model in our study is, however, in line with the updated 2020 draft NICE guidance that urges against this, except in specific circumstances.^2^ Some of the components of the CBT model in this study could be said to be included in the NICE guidance for example, ‘Addressing complex adjustment to diagnosis and acceptance of current functional limitations’. However, several of the key components of the model are not specified in the NICE guidance and require therapists to draw on a range of CBT concepts and techniques broader than those specific to CFS/ME (e.g., challenging high expectations of self and teaching tools for managing life with reduced resources). Participants in the study valued the ‘holistic’ (participant Susan) therapeutic approach, which allowed them to address a wide range of issues in their lives preventing them from accommodating and adapting to CFS/ME. Some components of the model go even broader than CBT and are more features of supportive therapies such as counselling (offering hope and a space to ‘lighten the load’). It is useful to note that for common mental health difficulties, it has been found that therapeutic alliance and therapist characteristics are key predictors of outcomes across client groups, for example see Budd and Hughes.^23^ Both of these may be important factors in this study, in which experienced therapists were adapting treatment to each participant. It is important to note that all participants in this study found it appropriate that the goal of therapy was positioned as better management of CFS/ME and quality of life rather than physical recovery. This model of CBT may not be suitable for service users who view the goal as recovery of pre‐CFS/ME activity levels.

### 4.4 Directions for future research

Research exploring psychological outcomes and processes in CBT for CFS/ME, aimed at better adapting to the illness rather than with the primary aim of increasing activity, is in its infancy. It may be appropriate to explore aspects of the model developed here through quantitative research: for instance, measuring changes in distress, acceptance and quality of life at intervals during this model of therapy. Mediation analyses might include self‐efficacy, sense of control, self‐compassion, perfectionism, ‘goal discrepancy’ and acceptance or denial of having a chronic illness or condition. It may also be useful to identify the active components of therapy using component analysis.^24^

Several theories implicate the body's stress hormonal systems as the basis of CFS/ME; it has been suggested for example that the condition arises from the same physiological processes as those that occur in ‘burnout’.^25^ It could therefore be hypothesised that the improvement in well‐being found in this study could lead to a reduction in fatigue in the long term. This requires further research. Multiple case research might examine how apparent improvements in daily living may lead to less experience of fatigue and activity restriction, and the points at which small successes and setbacks occur during the course of CBT, as well as outside of therapy.

Further research might also consider whether the current model could be extended to service users with more severe fatigue, for example, those who are homebound.

## 5 CONCLUSION

The study produced several novel findings. First, CBT aimed at improved management of CFS/ME, rather than increased activity, was viewed as acceptable by participants and appeared to alleviate distress about living with the condition. Second, this was not dependent on improvements in fatigue, and seemed to be facilitated by exploring and resolving issues around standards and identity and learning tools such as problem solving and pacing. Together, these seemed to facilitate participants adopting a more adaptive approach to daily life and becoming able to do more of what they valued. A common part of the therapeutic process was increased acceptance of the reality of having CFS/ME, leading to more adaptive coping. The results support the need for interventions addressing the above factors. However, it is unclear to what extent these apparent improvements in daily living may be maintained in the long term, and whether they may lead to less experience of fatigue and activity restriction. Further research is needed to test out the model suggested in this study.

## CONFLICT OF INTERESTS

The authors declare that there are no conflict of interests.

## Data Availability

Participants have not provided consent for full transcripts to be made available beyond this study.
